# Supplementary material for: On the Development of Phenol-Formaldehyde Resins Using a New Type of Lignin Extracted from Pine Wood with a Levulinic-Acid Based Solvent
Source: Molecules. 2022 Apr 29;27(9):2825. doi: 10.3390/molecules27092825 (PMC9105709; doi:10.3390/molecules27092825)
Supplement: Supplementary file 1 [file molecules-27-02825-s001.zip › molecules-1682472-supplementary.pdf]

On the development of phenol-formaldehyde resins using a new type of lignin extracted from pine wood with a levulinic-acid based solvent

Elodie Melro [1,*], Filipe E. Antunes [1,2], Artur J. M. Valente [1], Hugo Duarte [3], Anabela Romano [3], and Bruno Medronho [3,4],

[1] University of Coimbra, CQC, Department of Chemistry, Rua Larga, 3004-535 Coimbra, Portugal; fcea@qui.uc.pt (F.E.A.); avalente@ci.uc.pt (A.J.M.V)
[2] Science351 – Disruptive & Sustainable R&D Innovations, Instituto Pedro Nunes, Ed C, 3030-199 Coimbra, Portugal; filipe.antunes@science351.pt (F.E.A.)
[3] MED-Mediterranean Institute for Agriculture, Environment and Development, Universidade do Algarve, Faculdade de Ciências e Tecnologia, Campus de Gambelas, Ed. 8, 8005-139 Faro, Portugal; hugo.mm.drt@gmail.com (H.D.); aromano@ualg.pt (A.R.); bfmedronho@ualg.pt (B.M.)
[4] FSCN, Surface and Colloid Engineering, Mid Sweden University, SE-851 70 Sundsvall, Sweden
* Correspondence: elodie.melro@uc.pt

Supplementary information

Without lignin

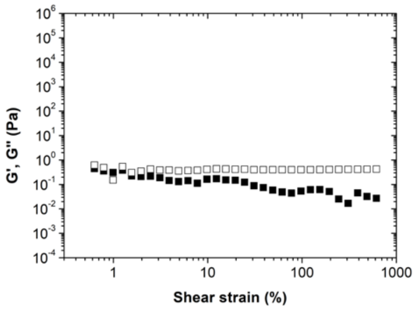

30 % Lignin	50 % Lignin

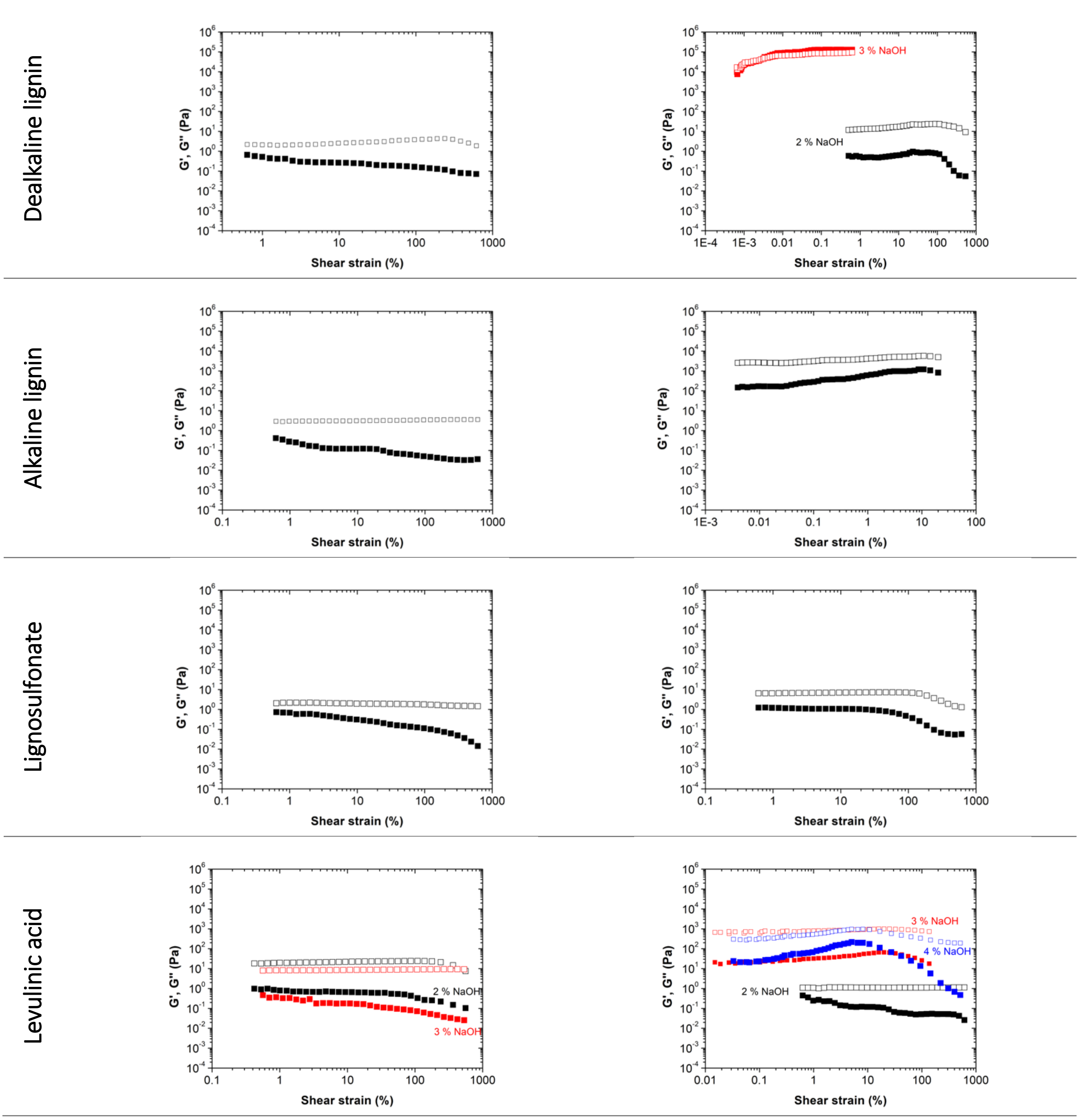

Figure S1. Strain amplitude sweep of resole resins, at 1 Hz and 20 °C. G′ is representing by full squares, while G′′ by empty squares. The different colors indicate the different NaOH concentration: 2 % - black, 3 % - red, 4 % - blue.

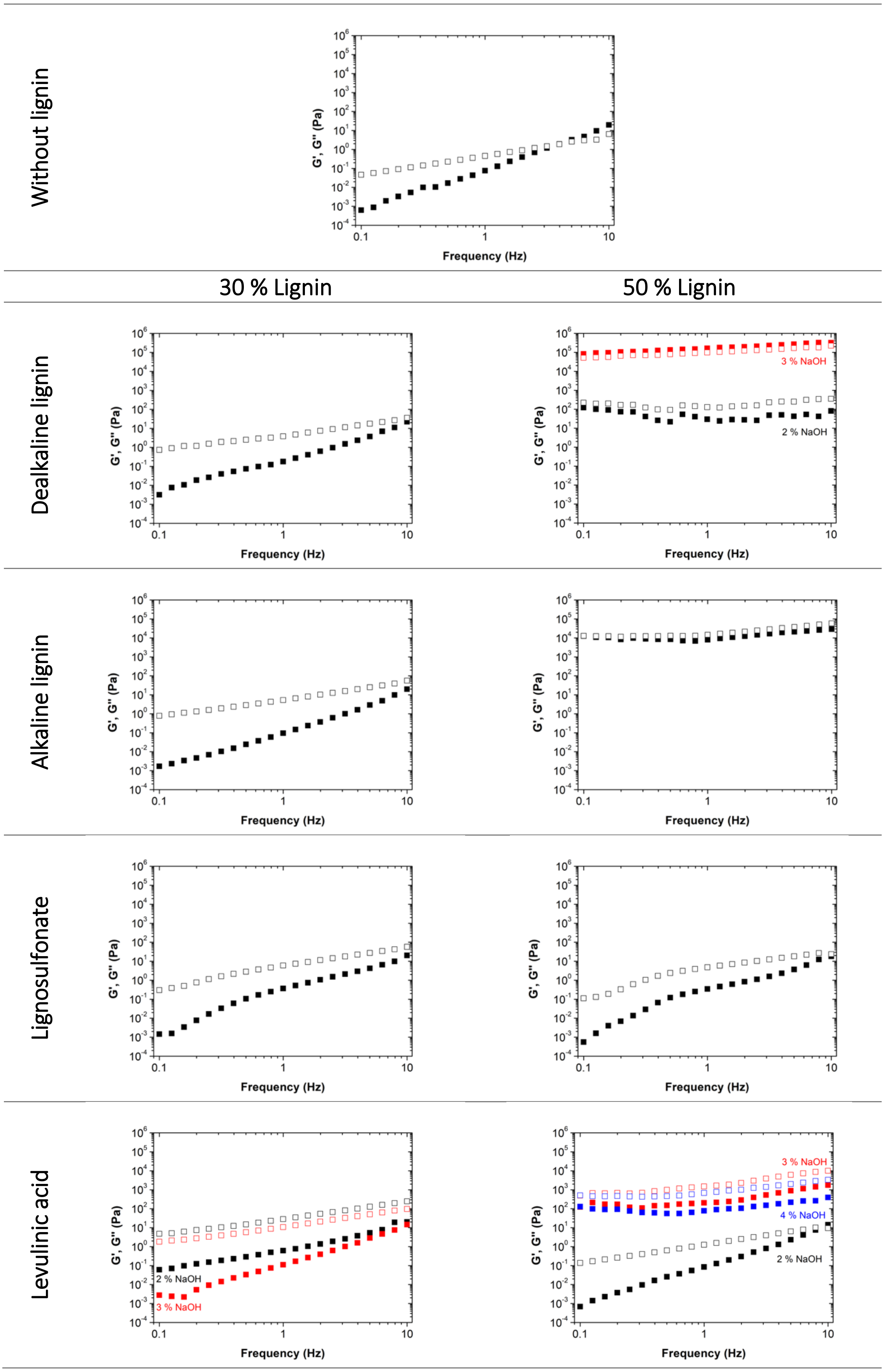

Figure S2. Frequency sweep of resole resins, at 5 Pa and 20 °C. G′ is representing by full squares, while G′′ by empty squares. The different colors indicate the different NaOH concentration: 2 % - black, 3 % - red, 4 % - blue.

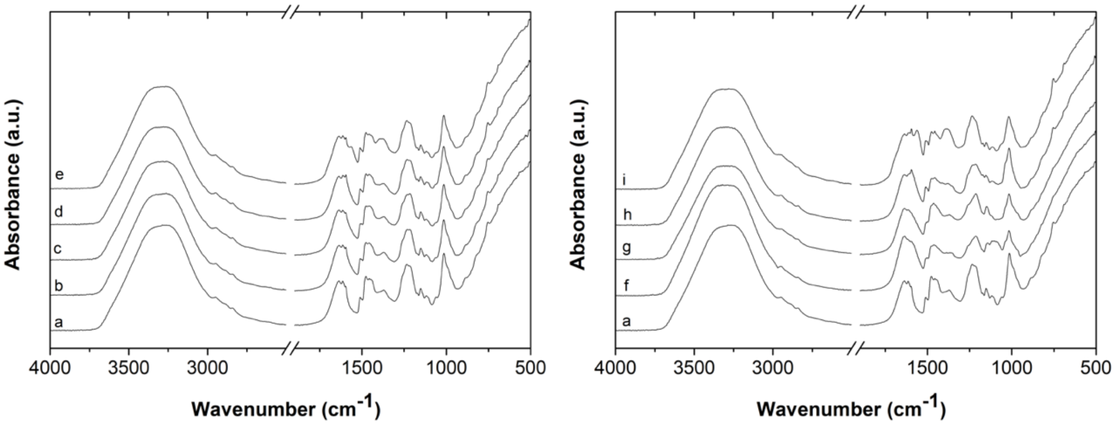

Figure S3. Normalized FTIR spectra of phenolic resins (a), 30 % (b-e) and 50 % (f-i) phenol replaced by lignin, using dealkaline lignin (b ,f), alkaline lignin (c, g), lignosulfonate (d, h) and lignin recovered from pine wood with levulinic acid (e, i), for optimized reins.
